# Salinity-Induced Cytosolic Alkaline Shifts in Arabidopsis Roots Require the SOS Pathway

**DOI:** 10.3390/ijms24043549

**Published:** 2023-02-10

**Authors:** Belén Rombolá-Caldentey, Zaida Andrés, Rainer Waadt, Francisco J. Quintero, Karin Schumacher, José M. Pardo

**Affiliations:** 1 Institute of Plant Biochemistry and Photosynthesis, Consejo Superior de Investigaciones Científicas, Universidad de Sevilla, 41092 Seville, Spain; 2 Centre for Organismal Studies, University of Heidelberg, 69120 Heidelberg, Germany

**Keywords:** salinity, cytosolic pH, salt-related signaling, SOS3/CBL4, roots, Arabidopsis

## Abstract

Plants have evolved elaborate mechanisms to sense, respond to and overcome the detrimental effects of high soil salinity. The role of calcium transients in salinity stress signaling is well established, but the physiological significance of concurrent salinity-induced changes in cytosolic pH remains largely undefined. Here, we analyzed the response of Arabidopsis roots expressing the genetically encoded ratiometric pH-sensor pHGFP fused to marker proteins for the recruitment of the sensor to the cytosolic side of the tonoplast (pHGFP-VTI11) and the plasma membrane (pHGFP-LTI6b). Salinity elicited a rapid alkalinization of cytosolic pH (pHcyt) in the meristematic and elongation zone of wild-type roots. The pH-shift near the plasma membrane preceded that at the tonoplast. In pH-maps transversal to the root axis, the epidermis and cortex had cells with a more alkaline pHcyt relative to cells in the stele in control conditions. Conversely, seedlings treated with 100 mM NaCl exhibited an increased pHcyt in cells of the vasculature relative to the external layers of the root, and this response occurred in both reporter lines. These pHcyt changes were substantially reduced in mutant roots lacking a functional SOS3/CBL4 protein, suggesting that the operation of the SOS pathway mediated the dynamics of pHcyt in response to salinity.

## 1. Introduction

Salinity stress imposes distinct challenges that affect plant physiology differently. The osmotic component of salinity is represented by high amounts of soluble ions in the root–soil interphase that reduce the water potential of the soil and hinder water uptake by the root. The osmotic component is sufficient to elicit a strong and rapid growth repression, mainly of aerial parts [1,2]. The ionic component of salinity stress results from the rising toxicity of sodium (Na^+^) ions accumulating gradually in plant tissues, although high concentrations of chloride (Cl^−^) may also become toxic to plants [3,4]. Na^+^ toxicity results from interference with potassium (K^+^) nutrition and disturbance of the K^+^/Na^+^ ratio of cells, which impairs plant physiology in several ways [2]. The strong hydration shell of Na^+^ ions may become chaotropic to macromolecules and thus Na^+^ can only partially replace K^+^ as a coordinating ion in certain enzymatic reactions [5]. However, Na^+^ is a suitable and energetically ”cheap” replacement for K^+^ as an osmotic agent, principally in vacuoles [6,7]. A third component of salinity stress, oxidative stress, results from enhanced production of reactive oxygen species (ROS) generated by both osmotic and ionic stresses to levels that are beyond the capacity for cellular anti-oxidant detoxification [8]. However, the salt-generated ROS signal has a role in the propagation of the salinity stress signal along the root and towards the shoot and in salt tolerance [8,9,10,11].

The salt overly sensitive (SOS) pathway is, by genetic criteria, the most important cellular mechanism involved in Na^+^ homeostasis and salt tolerance in several plant species, including halophytes [12]. The main components of the SOS pathway in Arabidopsis are the CALCINEURIN-B LIKE PROTEINS SOS3/CBL4, SCaBP8/CBL10 and CBL8, which bind calcium (Ca^2+^); the CBL-INTERACTING PROTEIN KINASES SOS2/CIPK24 and CIPK8 that are activated by the CBLs; and the plasma membrane Na^+^/H^+^ antiporter SOS1/NHX7 [12,13,14,15,16]. Upon sensing the Ca^2+^ spike triggered by salinity, the CBLs recruit the CIPKs to the plasma membrane to phosphorylate and activate SOS1 to extrude Na^+^ in exchange for apoplastic H^+^. Salt stress induces membrane depolarization, which enhances P-type H^+^-ATPase activity, and the proton motive force generated maintains SOS1 activity [17]. The SOS pathway operates in both roots and shoots with a difference in the Ca^2+^ sensor proteins. SOS3/CBL4 and CBL8 are preferentially expressed in roots, whereas CBL10 is more relevant in shoots [14,16]. Moreover, SOS3/CBL4 and CBL8 govern root Na^+^ tolerance at low and high intensities of salinity stress, respectively [16].

Plants have evolved elaborate mechanisms to sense, respond to, and overcome the detrimental effects of high soil salinity [18,19]. However, the molecular nature of initial perception of salt stress and the subsequent signal relay remain unclear [18,20,21]. Calcium is a universal secondary messenger [22]. Cytosolic free Ca^2+^ rapidly increases within seconds of plant exposure to NaCl [16,21,23], implying that salt sensor(s) are tightly coupled with stress-responsive Ca^2+^ channels [21]. Accordingly, the hyperosmolarity-gated Ca^2+^-permeable channel OSCA1 is involved in the perception of dehydrating conditions, and it has been suggested to act as an osmosensor in Arabidopsis [24]. A genetic screen in Arabidopsis identified the MONOCATION-INDUCED Ca^2+^ INCREASES 1 (*moca1*) mutant that is defective in salt-induced Ca^2+^ spikes [25]. MOCA1 is required for glycosyl inositol phosphorylceramide (GIPC) biosynthesis at the plasma membrane. Contrary to OSCA1, MOCA1 responds specifically to NaCl and not to a purely osmotic challenge. The unconventional Ca^2+^ channel ANNEXIN 1 mediates a Ca^2+^-permeable conductance in Arabidopsis roots that is activated by reactive oxygen species (ROS) and is important for the salt-responsive production of secondary roots [26]. Also in Arabidopsis, ANNEXIN 4 is involved in salinity-induced Ca^2+^ transients that specifically activate downstream components of the salt overly sensitive (SOS) pathway mediating resistance to Na^+^ ions [27]. Last, since salinity induces membrane depolarization, the depolarization-activated non-selective cation channels (DA-NSCC) are also plausible candidates to mediate Ca^2+^ influx.

The multiplicity of stimuli that can trigger Ca^2+^ transients as a common second messenger [22], together with the fact that Ca^2+^ is an essential macronutrient, prompts the question of how Ca^2+^ signaling can achieve output specificity [28]. Ca^2+^ transients can be further imbricated with and shaped by concurrent signals of different nature that assist signal decoding and propagation. Salinity produces ROS via the activation of the NADPH oxidases ROBHD and ROBHF [9,29,30]. ROBHD is necessary for the propagation of a ROS-assisted calcium wave that moves from the root apex upwards for long-distance root-to-shoot signaling in Arabidopsis seedlings [11]. The propagation of the ROS-Ca^2+^ wave relies on the activity of the tonoplast-localized TWO PORE CHANNEL 1 (TPC1) [10] that may release Ca^2+^ from the vacuole, which in turn activates NADPH oxidases at the plasma membrane via their Ca^2+^-binding EF-hands and/or through the phosphorylation by CBL1/9-CIPK26 complexes [31]. Newly produced apoplastic ROS by NADPH oxidases is suggested to trigger ROS-sensitive Ca^2+^ channels in the plasma membrane of neighboring cells, thereby propagating the self-sustaining ROS-assisted Ca^2+^-induced Ca^2+^-release wave [11].

Shifts in cytosolic pH (pHcyt) have also been invoked to play a signaling role supporting stimulus-specific outcomes [32,33,34,35]. Increases in cytosolic Ca^2+^ were accompanied by cytosolic acidification in response to several stimuli in both leaf and root cells of Arabidopsis [33]. Ca^2+^ and pHcyt were linked but not strictly coupled because different treatments produced specific “signatures” [33]. Simultaneous recording in Arabidopsis roots revealed that signaling intermediaries (glutamate, ATP, elicitor peptide, and glutathione disulfide) induced rapid and spatio-temporally overlapping cytosolic Ca^2+^, H^+^, and anion dynamics [34]. In tobacco, simultaneous ratiometric imaging of Ca^2+^ and pH indicated that Ca^2+^-dynamics lagged behind pH changes in guard cell closure and pollen tube growth [35]. These precedents support the notion that the pHcyt shifts might encode supplemental information additional to that of Ca^2+^ transients. However, the effect of salinity stress on pHcyt has been controversial, with conflicting reports of pHcyt increases, decreases, or no changes at all [32]. This inconsistency may result from the struggle to discriminate between early (signaling) and late (disturbed ion homeostasis) events triggered by salinity. In this regard, genetically encoded biosensors have emerged as powerful new tools allowing the monitoring in vivo and in real time of changes in the agonist signaling compound targeted by the biosensor in response to external and endogenous cues [36]. Therefore, we inspected the salinity-induced shifts of pHcyt in roots of *Arabidopsis thaliana* expressing the ratiometric pH-sensor pHGFP, a derivative of the pH-sensitive GFP ”pHluorin” specifically adapted for plant cells [37]. pHGFP was fused to markers for targeted localization to the tonoplast (VTI11) and the plasma membrane (LTI6b) to analyze the response in the vicinity of these cellular membranes. Moreover, we tested whether the salinity-induced changes in pHcyt were the direct consequence of disturbed ion homeostasis or were instead promoted by key signaling intermediaries in the salinity stress response.

Our results demonstrate that salt treatments produced a rapid cytosolic alkalinization in cells of the root segment comprising the meristematic and elongation zones. The increase in pHcyt was detected 2–3 min after the application of the NaCl treatment and no tendency to recover to the former resting state was observed during the 40 min time lapse of the experiment. Longer salinity treatments also resulted in the alkalinization of cells in the root stele around the early differentiation zone. Mutant roots lacking the SALT OVERTLY SENSITIVE 3 (SOS3) protein that were impaired in relaying the salinity-induced Ca^2+^ signal were also less responsive in generating salt-induced pH-shifts than the wild-type root, suggesting that full-fledged salt-induced cytosolic alkalinization requires proper signaling through the SOS pathway.

## 2. Results

### 2.1. In Vivo Measurement of Cytosolic pH with Genetically Encoded Sensors

For this study, we selected the genetically encoded pH-sensor pHGFP, which was developed for ratiometric pH imaging in Arabidopsis roots in the range between pH 5.5 to 7.5 [37]. Moreover, to monitor cytosolic pH dynamics near cellular membranes, the free cytosolic pHGFP was translationally fused to the protein markers VTI11 and LTI6b, thereby targeting the recombinant protein to the cytosolic side of the tonoplast (pHGFP-VTI11) or the plasma membrane (pHGFP-LTI6b) [38,39]. VTI11 is a v-SNARE protein involved in vacuole fusion [40,41]. LTI6b is a small cold-inducible protein that recruits pHGFP to the plasma membrane [39]. Constructs for the expression of pHGFP-VTI11 and pHGFP-LTI6b were transformed independently into *A. thaliana* Col-0, and homozygous lines expressing the fusion proteins were obtained. Localization of the pHGFP-VTI11 and pHGFP-LTI6b fusions to the tonoplast and the plasma membrane has been demonstrated elsewhere [38,39].

First, we tested whether the lines expressing pHGFP-VTI11 and pHGFP-LTI6b were sensitive to externally imposed cytosolic pH variations. A graphical representation of the experimental procedure is given in Supplemental Figure S1. *A. thaliana* Col-0 seedlings expressing the pHGFP reporters were grown vertically onto plates with LAK medium and were subsequently transferred to microscope dishes with LAK medium supplemented with 0.7% low melting point agarose. Before imaging, the seedlings were placed horizontally, 90 μL of liquid LAK medium was added onto the roots, and seedlings were allowed to recover for 40–60 min. Next, the pHGFP reporter was excited with 405 and 488 nm light wavelengths, and emission was detected in a 500–550 range. Ratiometric fluorescence data are given as the emission ratios (R) after excitation with 405 and 488 nm light wavelengths. Frames were acquired every 6 s. After 4 min of fluorescence baseline recordings, 10 µL of solutions imposing different treatments were added on the side of the root, and imaging acquisition continued for 40 min. Maximal treatment-induced pH variations were detected in the region comprising the meristematic and elongation zones (MEZ segment; 750 µm upwards from the root tip). The fluorescence data were compiled graphically as heat-maps representing pH variations along the MEZ axis over time.

**Figure 1:**
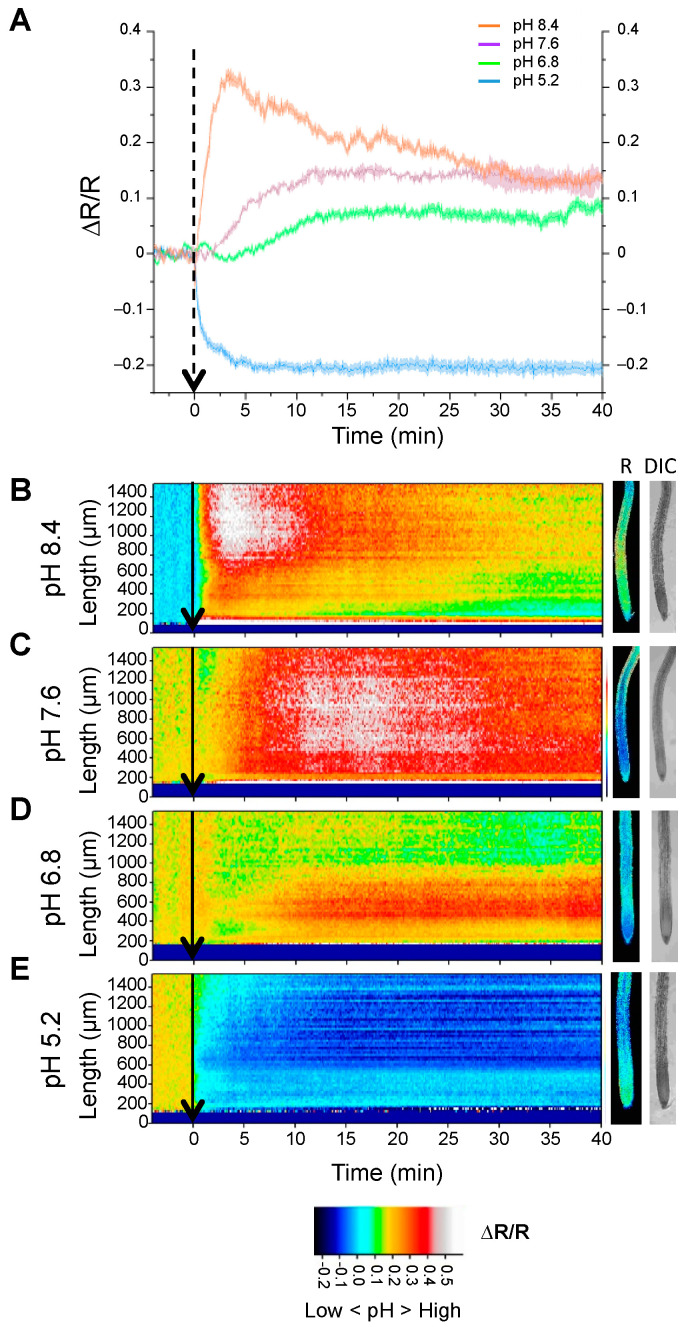
Response of pHGFP-VTI11 to external pH. Measurement of variations in pHcyt sensed by the pHGFP-VTI11 reporter in Arabidopsis thaliana Col-0 seedlings. (**A**) Normalized emission ratio (ΔR/R) changes, presented as means ± SEM. Values derive from the quantifications of ΔR/R in the MEZ zone of at least two independent experiments per treatment. (**B**–**E**) Heat-maps of normalized fluorescence data derived from 64 adjacent regions (268.2 × 24.2 µm) according to the length scale shown. Arrows indicate the beginning of the treatment. Pictures on the right show fluorescence emission ratio (R) and bright-field (DIC) images of roots at time point 35 min. Below, frames with false-color calibration bars of the heat map. Heat-maps and root pictures are representative of at least 2 independent experiments for each treatment.

Seedlings expressing the tonoplast-localized pHGFP-VTI11 reporter were treated with 10 mM BTP-MES/HEPES buffers adjusted to different pH (5.2, 6.8, 7.6, and 8.4). The variation in pHcyt was immediately detected when the buffers with more extreme pH were applied (Figure 1). With buffers near neutrality (pH 7.6 and 6.8), the detection of pHcyt variations was delayed relative to extreme treatments. The results show that the addition of acidic buffer (pH 5.2) decreased the emission ratio, indicating an acidification of the cytosol. The opposite occurred when a basic buffer was added and the emission ratio increased, indicating alkalization of the cytosol.

**Figure 2:**
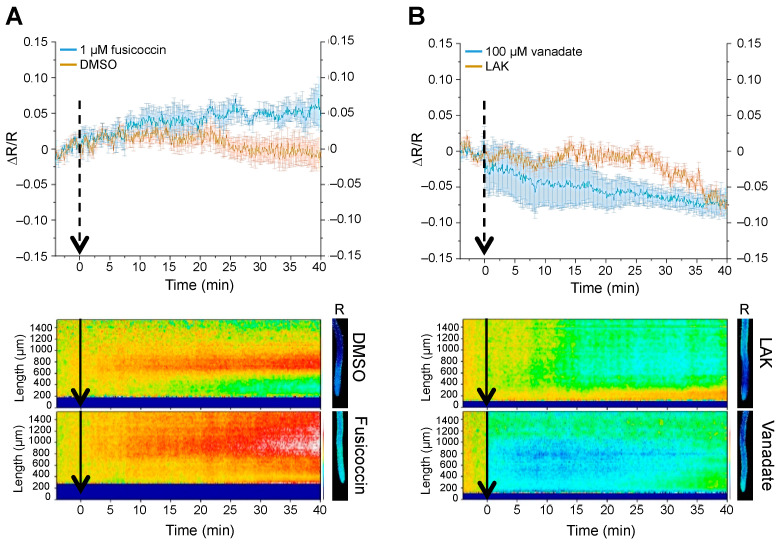
Response of pHGFP-LTI6b to altered activity of the PM H^+^-pump. pHGFP-LTI6b response to activation of the plasma membrane H^+^-ATPase by fusicoccin (**A**) and inhibition of the plasma membrane H^+^-ATPase by vanadate (**B**). Data in plots are the quantifications of ΔR/R in the MEZ zone. The plots represent the mean of 2 independent experiments ± SEM. Arrows indicate the time the treatment started. Heat-maps below show representative experiments. Pictures on the right show fluorescence emission ratio (R) images of representative roots used at the 35 min time point. Heat-maps and root pictures are representative of at least 2 independent experiments for each treatment.

In a second tier of experiments, we determined whether the pHGFP reporter was sensitive to cytosolic pH variations imposed from within the cell. To that end, pHGFP-LTI6b seedlings reporting pHcyt changes in the proximities of the plasma membrane were treated with the fungal toxin fusicoccin (FC). This compound binds to the complex formed by the plasma membrane H^+^-ATPase and the 14–3-3 proteins, stabilizing the interaction. As a result, the pump is permanently activated [42,43] and the enhanced extrusion of H^+^ from the cytosol should be reported by pHGFP-LTI6B as pHcyt increases. As expected, the pHGFP-LTI6b reporter detected alkalinized pHcyt after the FC addition relative to the mock treatment with DMSO (Figure 2A). In the converse experiment, pHGFP-LTI6b seedlings were treated with the plasma membrane H^+^-ATPase inhibitor vanadate [44]. In this case, the pump inhibitor caused cytoplasmic acidification, which is coherent with impaired translocation of H^+^ mediated by the H^+^-ATPase (Figure 2B). From these results, we concluded that the Arabidopsis lines expressing the constructs pHGFP-VTI11 and pHGFP-LTI6b are reliable biological tools to study cytosolic pH variations in the physiological range for plant cells.

### 2.2. Cytosolic pH Variations near the Tonoplast and Plasma Membranes under Salt Stress

**Figure 3:**
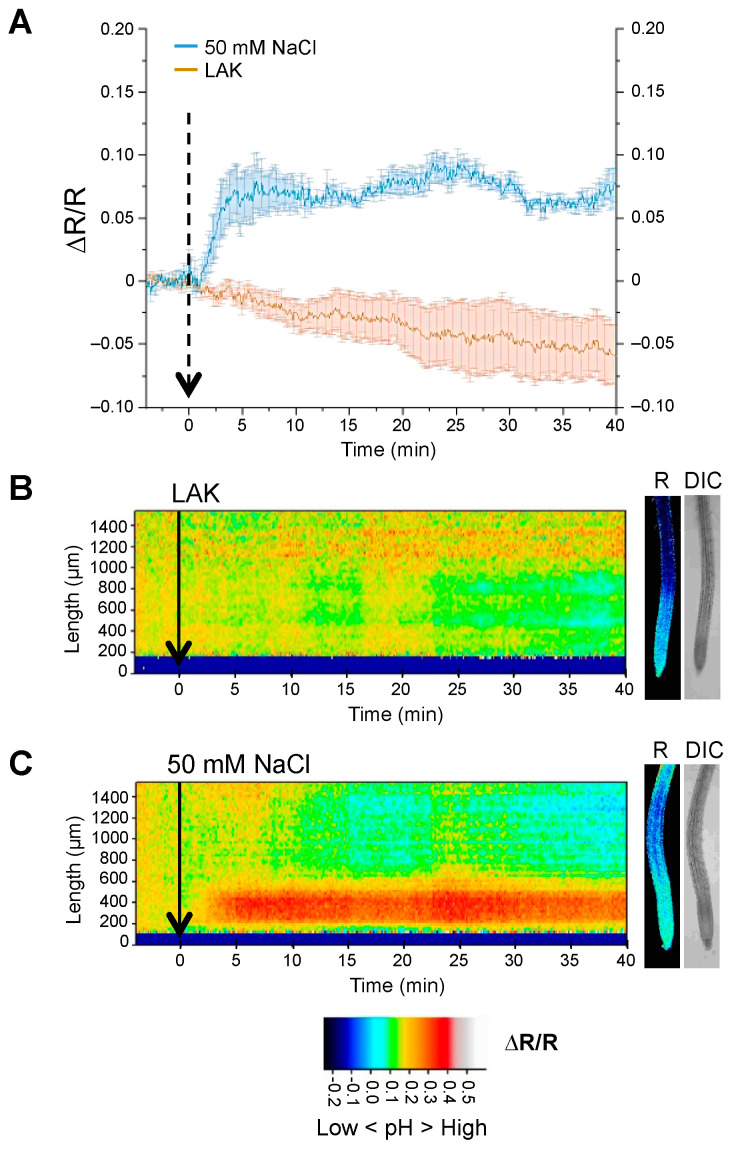
Cytosolic pH variations under salt stress near the plasma membrane. Seedlings of the Arabidopsis line expressing pHGFP-LTI6b were treated with either LAK medium (**B**) or LAK supplemented with 50 mM NaCl (**C**). (**A**) Normalized emission ratio (ΔR/R) changes of pHGFP-LTI6b with and without salt treatment; lines indicate the mean of emission ratio of the MEZ segment ± SEM. (**B**,**C**) Heat-maps of normalized data derived from 64 adjacent regions (268.2 × 24.2 µm). Small pictures at the right of the heat-maps show fluorescence emission ratio (R) and bright-field (DIC) images of representative at 35 min time point. Arrows in the three panels indicate the beginning of the treatment. Heat-maps and roots pictures correspond to a representative experiment from 5 to 6 independent experiments.

The salt stress response in *A. thaliana* roots modifies the activity of various ion transporters at the plasma membrane that include P-type H^+^-ATPases, K^+^ transporters and channels, and the Na^+^/H^+^ exchanger SOS1 that is critically important for salt tolerance [12,45]. Alteration of these ion fluxes under salinity stress could translate into pHcyt dynamics as part of the salinity stress response or as a consequence of disturbed ion homeostasis. To address this point, seedlings of the pHGFP-LTI6b reporter line were treated with 50 mM NaCl. The salt treatment produced an alkalization of pHcyt in root cells in comparison to the roots treated with the control medium. As previously detected, the main response was observed at the MEZ segment (Figure 3). The increase in pHcyt was detected within 1–2 min after the application of the NaCl treatment. Notably, no tendency to restore the former resting state during the elapsed time of the experiment (40 min) was observed (Figure 3 and Supplemental Figure S2).

**Figure 4:**
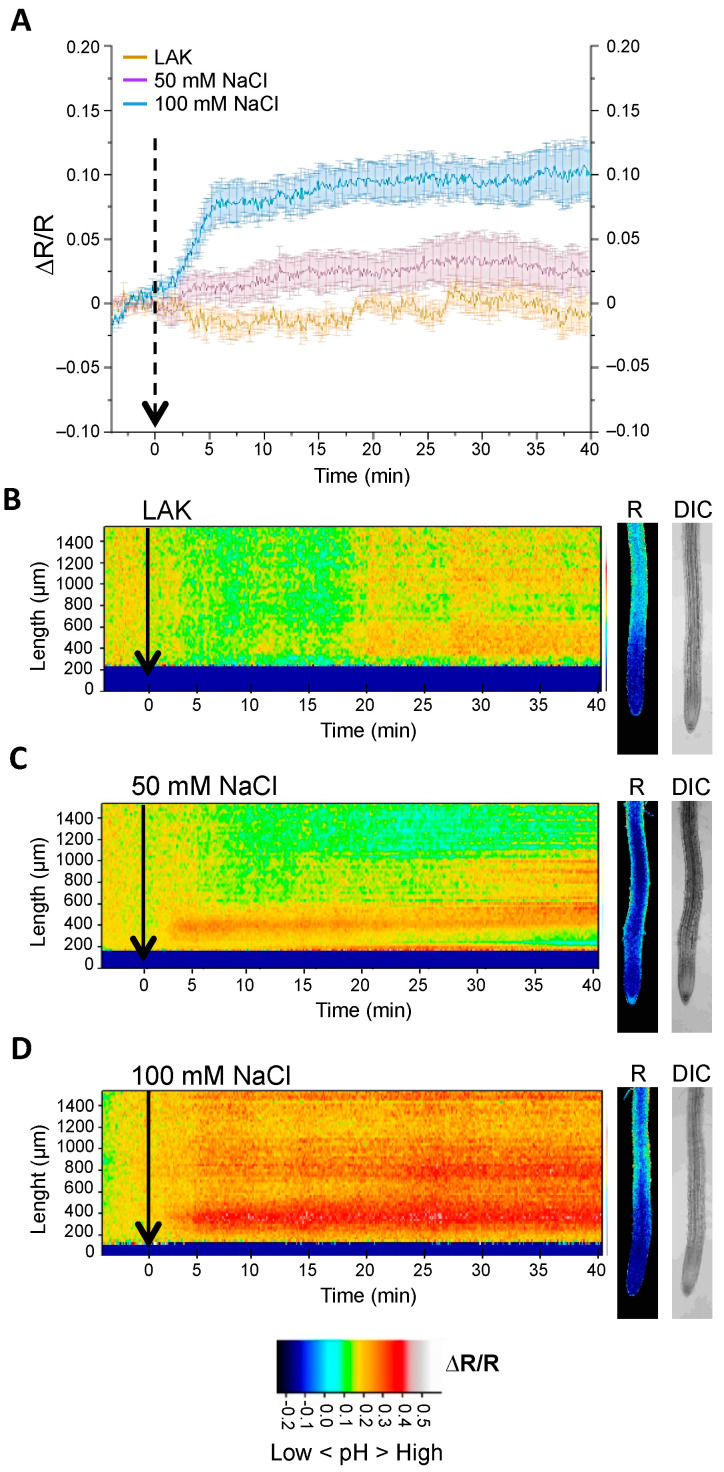
Cytosolic pH variations under salt stress near the vacuolar membrane. Seedlings of the Arabidopsis line expressing the pH sensor pHGFP-VTI11 were treated with either LAK medium 1 mM K^+^ (**B**) or the same medium supplemented with 50 mM NaCl (**C**) or 100 mM NaCl (**D**). (**A**) Normalized emission ratio (ΔR/R) changes of pHGFP-VTI11 with or without NaCl treatment; lines indicate the mean of emission ratio of the MEZ sector ± SEM from 5 to 6 seedlings. (**B**,**D**) Heat-maps of normalized data derived from 64 adjacent regions (268.2 × 24.2 µm) according to the position scale on the left. On the right are shown fluorescence at time point 35 min and bright-field (DIC) images. Arrows indicate the beginning of the treatment. Heat-maps and roots pictures correspond to a representative experiment from 5 to 6 independent experiments.

Ion fluxes at the tonoplast are thought to preserve and help restoring the cytosolic pH and ion homeostasis of the cell [46,47,48,49]. Moreover, the main physiological activity under salinity stress is Na^+^ compartmentalization into the vacuole, which proceeds through Na^+^/H^+^ exchange energized by proton pumps in the tonoplast [46,50]. Therefore, the pHGFP-VTI11 reporter line was treated with different concentrations of NaCl to determine how the salt stress affected cytosolic pH in the vicinity of the vacuolar membrane and if the activity of transport proteins in this organelle elicited a different pHcyt dynamics compared to the plasma membrane. Changes in pHcyt were recorded after treatment of pHGFP-VTI11 seedlings with two different NaCl concentrations, 50 mM and 100 mM (Figure 4). The pHcyt response was dose-dependent, with a significant yet modest cytosolic alkalinization in response to 50 mM NaCl that was also of lower amplitude compared to the plasma membrane (see Figure 3). Treatment with 100 mM NaCl produced a robust response comparable in magnitude to the pHcyt shift attained with 50 mM NaCl in pHGFP-LTI6b seedlings, but slightly delayed in time relative to pHGFP-LTI6b seedlings (ca. 1 min when the ∆R/R curves were compared). The 100 mM NaCl treatment induced an increased pH along the whole imaged root, but, again, it was more intense in the MEZ region (Supplemental Figure S2).

### 2.3. Changes in Cytosolic pH in Whole Roots under Salt Stress

From the above results, it was concluded that salt stress produced an increase in the cytosolic pH of the root cells at the MEZ root segment. However, not much information about pHcyt variations in different root tissues could be obtained from that imaging protocol. To investigate how the pHcyt of the entire root varied under salt stress, pH-maps of the complete seedling were generated according to [51]. For this, 3–4-day-old seedlings expressing the pHGFP-LTI6b and pHGFP-VTI11 reporters were grown vertically in LAK plates, transferred to new dishes with the same medium supplemented or not with NaCl, and left to recover overnight in a growth chamber (Supplemental Figure S1). The day after, seedlings were imaged directly onto the dish. Images obtained from these measurements were processed with Fiji software to obtain quantitative pH data. In these experiments, the emission ratio was measured in separate segments corresponding to four different phenological zones of the root, namely the elongation zone S1 (equivalent to MEZ in previous experiments), a transition zone S2 in which the vascular bundle and root hairs were starting to differentiate, a differentiated zone S3 is which the vascular bundle was fully formed, and the mature zone S4 near the cotyledons (Supplemental Figure S3).

**Figure 5:**
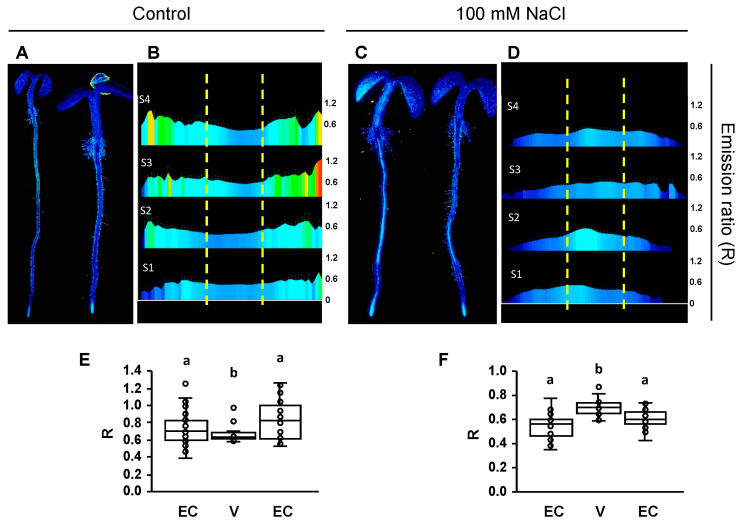
pHcyt maps of whole seedlings expressing the pH-sensor pHGFP-LTI6b. Fluorescence emission ratios after sequential excitation at 405 and 488 nm (R) of pHGFP-LTI6b in 4-day-old seedlings 16 h after being transferred to LAK media (**A**,**B**,**E**) or supplemented with 100 mM NaCl (**C**,**D**,**F**). (**A**,**C**) Representative seedlings grown in LAK medium (**A**) or LAK supplemented with NaCl (**C**). (**B**,**D**) Profile of root sectors showing average emission ratios (R) from at least 6–8 seedlings per treatment; the root zones S1 to S4 are named as in Supplemental Figure S3; the scales of R values are shown on the right; yellow lines indicate the division used to analyze the vasculature (center) and the epidermis and cortex (flanks). (**E**,**F**) Box plots indicating the mean of the emission ratios (R) in different tissues for each condition. The mean was obtained from the aggregated sectors S1–S4 shown in the images above. EC: epidermis and cortex; V: vasculature. Different letters indicate groups with significantly different values according to Tukey HSD test at *p* < 0.05.

**Figure 6:**
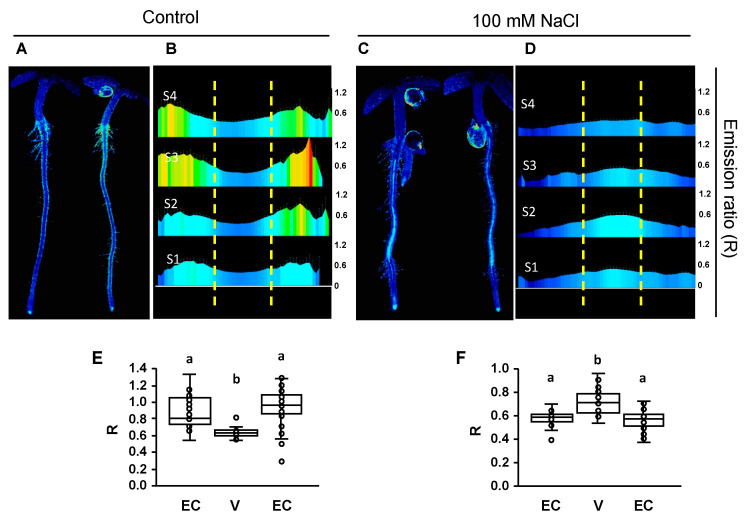
pHcyt maps of whole seedlings expressing the pH-sensor pHGFP-VTI11. Fluorescence emission ratios after sequential excitation at 405 and 488 nm (R) of pHGFP-VTI11 in 4-day-old seedlings 16 h after being transferred to LAK media (**A**,**B**,**E**) or LAK supplemented with 100 mM NaCl (**C**,**D**,**F**). (**A**,**C**) Representative seedlings grown in LAK medium (**A**) or LAK supplemented with NaCl (**C**). (**B**,**D**) Profile of root sectors showing average emission ratios (R) from at least 6 to 8 seedlings per treatment; the root zones S1 to S4 are named as in Supplemental Figure S3; the scales of R values are shown on the right; yellow lines indicate the division used to analyze the vasculature (center) and the epidermis and cortex (flanks). (**E**,**F**) Box plots indicating the mean of the emission ratios (R) in different tissues for each condition. The mean was obtained from the aggregated sectors S1–S4 shown in the images above. EC: epidermis and cortex; V: vasculature. Different letters indicate groups with significantly different values according to Tukey HSD test at *p* < 0.05.

Fluorescence imaging found substantial differences between seedlings in the saline medium and the mock treatment with regular LAK medium (Figure 5 and Figure 6). From pH-maps transversal to the root axis of seedlings grown in normal conditions, it appeared that epidermis and cortex had cells with a more alkaline pHcyt relative to cells in the stele. This pattern changed towards a higher pH in the vasculature and a lower pH in the epidermis and cortex in response to salt stress. Although the whole root exhibited a similar pattern, the fluorescence data were collected from segments S1–S4 of at least 6–8 seedlings, and a pHcyt profile for each of these segments was created (Figure 5 and Figure 6). The difference in pHcyt between root cell layers was maximal in section S4 and less in S1 in pHGFP-LTI6b seedlings, whereas pHGFP-VTI11 seedlings exhibited a more consistent pattern between sections. Conversely, seedlings treated with 100 mM NaCl exhibited an increased pHcyt in cells of the vasculature relative to the external layers of the root, and this response occurred in both reporter lines (Figure 5 and Figure 6). In this case, maximal alkalinization occurred in stellar cells of section S2 (early differentiation stage of vascular bundle and root hairs). The statistical analyses (Tukey HSD test, *p* < 0.05) indicated that the differences of variation observed between the vasculature (V) and the epidermis and cortex (EC) of seedlings in control condition or under salt treatment were significant (Figure 5E,F and Figure 6E,F).

### 2.4. Cytosolic pH Shifts under Compromised Salt-Stress Sensing

The SOS pathway is one of the main mechanisms that plant cells activate under salt stress in order to extrude Na^+^ from the cytosol, which is linked to the re-entry of extracellular H^+^ via the plasma membrane Na^+^/H^+^ exchanger SOS1 [52,53,54]. This pathway is dependent on the activity of the Ca^2+^-sensor proteins SOS3/CBL4, SCaBP8/CBL10, and CBL8 [13,14,16]. Among these CBLs, SOS3/CBL4 is particularly relevant in the early root response to salinity [16].

**Figure 7:**
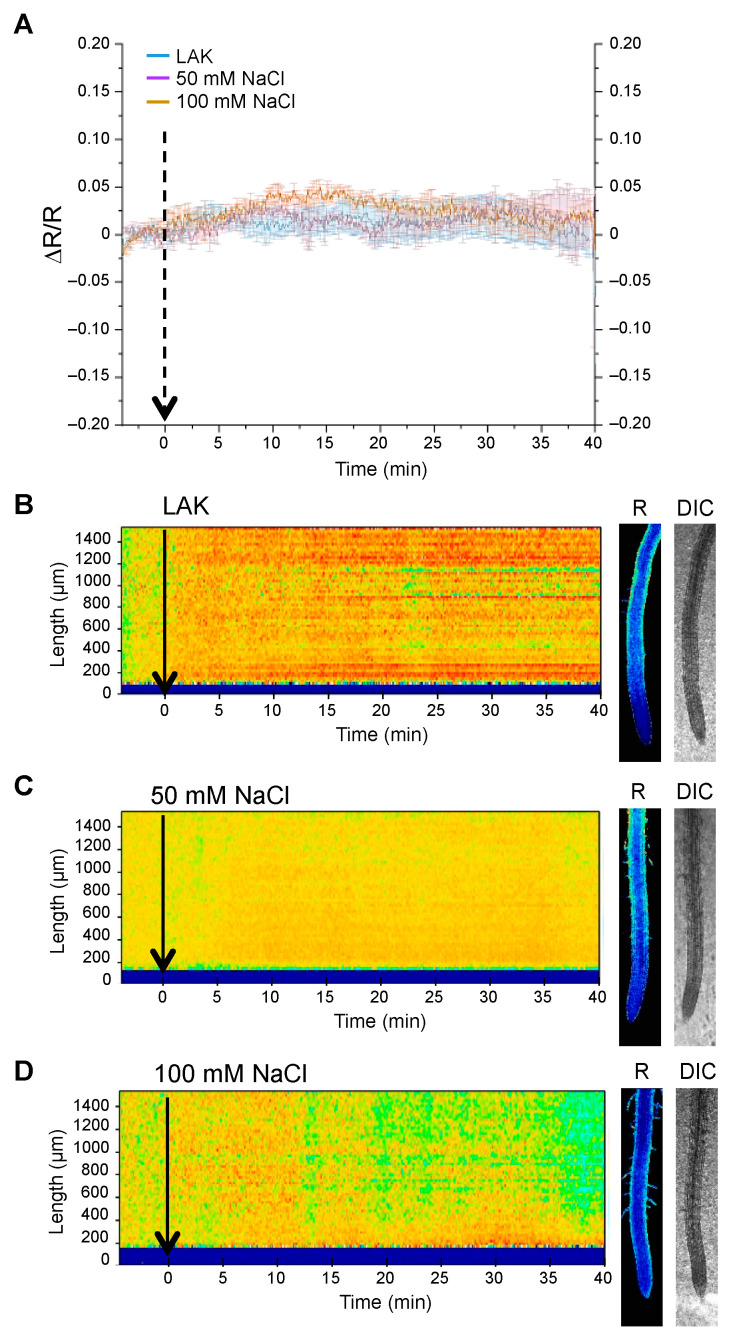
Cytosolic pH variations under salt stress reported by pHGFP-LTI6b in the *sos3* mutant. Seedlings of the *sos3-1* mutant expressing the pH-sensor pHGFP-LTI6b were treated with either LAK medium (**B**) or the same medium supplemented with 50 mM NaCl (**C**) or 100 mM NaCl (**D**) and the variations in pHcyt were measured. (**A**) Normalized emission ratio (ΔR/R); lines indicate the mean of emission ratio of the MEZ sector ± SEM from 5 to 6 seedlings. (**B**–**D**) Heat-maps of normalized data derived from 64 adjacent sections (268.2 × 24.2 μm) according to the position scale on the left. On the right are shown fluorescence (R) and bright-field (DIC) images at time point 35 min. Heat-maps and root micrographs correspond to representative experiments. Arrows in all panels indicate the time the treatment started.

To analyze the contribution of the SOS3 sensor protein to salt stress response, pHcyt variations under salt stress conditions were measured in the *sos3-1* mutant in comparison with the wild type. The *sos3-1* mutant expresses a dysfunctional SOS3 protein with a defective EF-hand calcium-binding site, making it unable to activate SOS2 [55]. Because SOS3 targets the protein kinase SOS2/CIPK24 to the plasma membrane for the activation of the SOS1 Na^+^/H^+^ exchanger [13,56], the *sos3-1* mutant was transformed with the pHGFP-LTI6b reporter localized at the plasma membrane. A first approach was aimed to compare the most salt-responsive root zone, the MEZ segment, of the *sos3-1* mutant and wild-type seedlings. As shown in Figure 7, no significant differences in pHcyt were observed when the *sos3-1* mutant expressing pHGFP-LTI6b was treated with 50 and 100 mM NaCl solution relative to non-treated seedlings, in contrast to the salt-dependent response observed with wild-type seedlings expressing the reporters pHGFP-LTI6b and pHGFP-VTI11 (Figure 2 and Figure 3). These results indicate that the meristem and elongation zone (MEZ) of *sos3-1* roots is poorly responsive to salinity stress.

**Figure 8:**
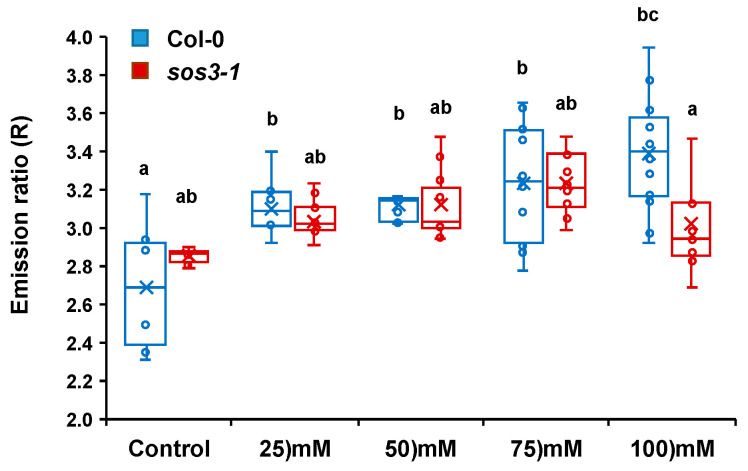
Cytosolic pH variations under salt stress reported by pHGFP-LTI6b in wild-type Col-0 and *sos3* mutant seedlings. Seedlings expressing the pH-sensor pHGFP-LTI6b were treated with either LAK medium or the same medium supplemented with increasing concentrations of NaCl as indicated, and the variations of pHcyt were measured. Handling and measurements followed the One Shot per Root method. Fluorescence emission ratios (R) of pHGFP-LTI6b with or without treatment for each genotype are represented. The box plot shows individual data points. Outliers are not shown but were included for statistical analysis. The ”x” symbol represents the means. Different letters indicate groups with significantly different values according to Tukey HSD test at *p* < 0.05. The experiment was repeated twice with similar results.

Inspection of whole seedlings evidenced that other root sectors besides MEZ had differential responses to NaCl in wild-type and *sos3-1* seedlings. To quantify these differences with improved statistical robustness, the whole-root imaging protocol was modified. The alternative approach, termed the One Shot per Root method (see Materials and Methods for details), collected fluorescence data resolved in space and time, allowing the imaging of 8–9 seedlings per genotype and treatment while minimizing sample-to-sample experimental noise (Supplemental Figure S1). The wild-type Col-0 and mutant *sos3-1* lines expressing the pHGFP-LTI6b reporter were treated with increasing concentrations of NaCl (25, 50, 75 and 100 mM NaCl), imaged using the One Shot per Root method, and the imaging data was statistically analyzed. The results from these analyses (Figure 8) show that, although both genotypes displayed the same tendency towards an alkaline shift of pHcyt in response to increasing NaCl concentrations, differences between whole roots of the Col-0 line and *sos3-1* mutant were also observed. Firstly, the emission ratios suggest that *sos3-1* seedlings had a more alkaline pHcyt than Col-0 under control non-saline conditions. Secondly, at 75 mM NaCl the emission ratios were more alkaline in Col-0 than in *sos3-1* seedlings. Together, these differences signify that the dynamic range of the pHGFP-LTI6b response was greater in Col-0 than in the *sos3-1* mutant. At the highest concentration used, 100 mM NaCl, both genotypes exhibited less alkalinization than at 75 mM NaCl. Because the decline was more pronounced in the salt-sensitive *sos3-1* seedlings, this could reflect salt-induced toxicity and impaired physiological response in these experimental conditions. Together, these data imply that roots of *sos3-1* mutant seedlings are less responsive to salt-induced pH-shifts than the wild type.

**Figure 9:**
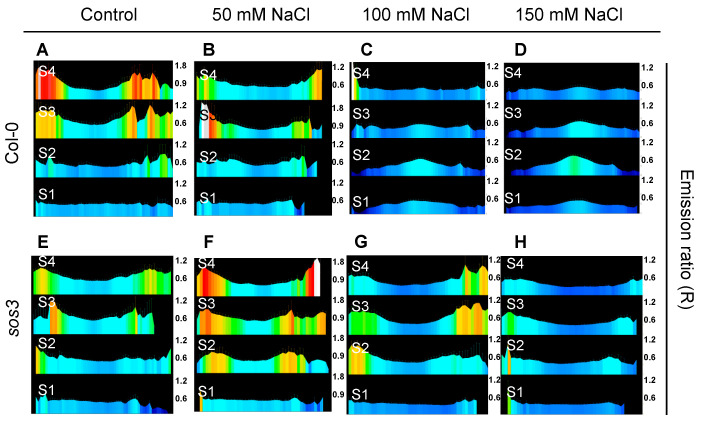
Root pH-maps under salt stress of wild-type Col-0 and *sos3* seedlings. Fluorescence emission ratios (R) of pHGFP-LTI6b in 4-day-old seedlings 24 h after being transferred to LAK medium supplemented with the indicated NaCl concentrations. (**A**–**H**) Panels present the fluorescence emission ratios (R) of cross-sections of the root zones defined in Supplemental Figure S3 of 5–6 seedlings for each segment. The scales of the R values are shown on the right of each panel.

To test if different root tissues exhibited a differential response to NaCl depending on the genotype, pH-maps of whole seedlings were generated and sectors S1 to S4 were analyzed transversally (Figure 9). The pHGFP-LTI6b reporter in wild-type Col-0 seedlings exhibited the same pHcyt variations as previously described for the tonoplast and plasma membrane reporters (Figure 5 and Figure 6). However, the vasculature of the *sos3-1* mutant did not show the characteristic cytoplasmic alkalinization observed in the wild type independently of the salt concentration applied (Figure 9 and Supplemental Figure S4).

**Figure 10:**
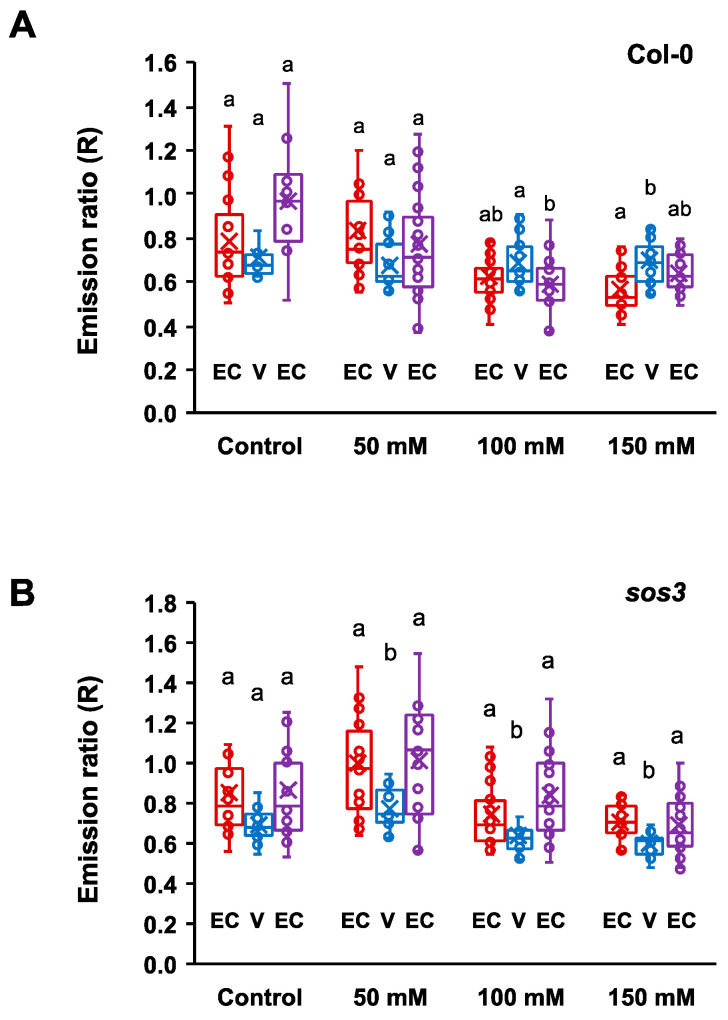
Cytosolic pH variation across the root sections of wild-type seedlings and the *sos3* mutant. Box plots represent the mean fluorescence emission ratios (R) of pHGFP-LTI6b in different tissues for each condition and genotype. (**A**) Col-0, (**B**) *sos3-1*. EC: epidermis and cortex; V: vasculature. Values were plotted from root segments S1 to S4 as defined in Supplementary Figure S3. Means are marked with symbol “x”. Outlier points are not shown but were included for statistical analyses. Different letters indicate groups with significantly different means for each genotype according to the Tukey HSD test at *p* < 0.05.

The emission ratio (R) data obtained for each seedling in S1 to S4 were aggregated and compared according to the different root tissues as described above, i.e., epidermis plus cortex at both sides of the vasculature (EC1 and EC2) and the vascular bundle (V). The mean emission ratio for each tissue was calculated and plotted (Figure 10). Results show that at 50 mM NaCl, the difference in emission ratio between the vasculature (V) and the surrounding tissues (EC) was statistically more significant in *sos3-1*, in contrast to the wild type, because the EC tissues were more alkaline in the mutant. Strikingly, the inverted pattern typical in wild-type seedlings at 100 and 150 mM NaCl, in which the vasculature became more alkaline than the surrounding tissues, was not observed in the *sos3-1* mutant (Figure 10). Together, these results point to SOS3 as an important signaling effector in the salt stress response affecting the alterations of pHcyt in root cells resulting from salinity stress.

## 3. Discussion

Plants are exposed to a multitude of environmental inputs and stressors to which they respond by activating signaling pathways that start with stimulus perception. Among the several mechanisms known to convey salt tolerance, the ability to extrude cytosolic Na^+^ is strongly correlated with salinity stress tolerance in different plant species [18,57,58]. This exclusion is mediated by the plasma membrane (PM) Na^+^/H^+^ antiporter encoded by *SALT OVERLY SENSITIVE 1* (*SOS1*) gene and driven by the proton gradient generated by plasma membrane H^+^-ATPases [12,52,54,59].

Changes in pH in the cytoplasm and the lumen of organelles are now widely accepted as a signaling mechanism in plant cells [33,34,60]. Even though cytosolic pH is tightly regulated, alterations in the activity of H^+^-pumps and H^+^-energized secondary transporters in different tissues and membranes of the cell may produce local and transient pH changes that could act as a signal. The luminal pHs of endomembranes have already been obtained through the use of genetically encoded pH indicators [60,61,62]. Generating a pH-map of *A. thaliana* roots under salinity would allow a better understanding of how the cytosolic pH is affected upon salt stress. Moreover, the specific detection of these pH variations in the surroundings of the different cellular membranes may enable the study of how the physiological activity of the proteins in the membranes of each organelle affect the cytosolic pH. To that end, *A. thaliana* Col-0 plants were generated that express the genetically encoded pH-sensor pHGFP, which was translationally fused to protein markers that localize either at the plasma membrane (LTI6b) or the tonoplast (VTI11) [38,39]. The pH reporting capacities of pHGFP-VTI11 and pHGFP-LTI6b were validated using externally imposed pH challenges and applying the H^+^-pump inhibitor vanadate or the activating drug fusicoccin. To document cytosolic pH dynamics in response to salinity stress, the Arabidopsis lines expressing pHGFP-VTI11 and pHGFP-L16b were challenged with increasing concentrations of NaCl.

Previous reports addressing the changes in pHcyt upon saline (NaCl) treatment have yielded inconsistent results. Salt-induced cytoplasmic alkalinization [63,64], acidification [65], no significant response [66,67], and even the opposite dynamics of pHcyt depending on the contrasting salt tolerance of rice cultivars [68] or treatment length [69] have been described. However, these reports were carried out with internalized fluorescent chemical probes, protoplasts, in single-cell systems or leaves, or were restricted to predefined root segments. In our experimental setup, we used whole seedlings expressing a ratiometric genetically encoded pH-sensor whose accurate response to externally imposed pH-shifts was experimentally assessed (Figure 1 and Figure 2). The pH-maps of salt-treated seedlings show a substantive alkalinization of a group of cells in the meristematic and early differentiation zone (MEZ). Additionally, a differential alkalinization in the vasculature relative to the epidermal and cortical tissues was found. However, the lack of a reliable calibration curve in fully organized tissues to relate changes in emission rates to actual pHcyt values did not allow for discriminating whether the relative acidification of the cortex and epidermis corresponded to an actual decrease in pHcyt compared to the seedlings in normal conditions, or just relative to the vasculature in the same conditions.

**Figure 11:**
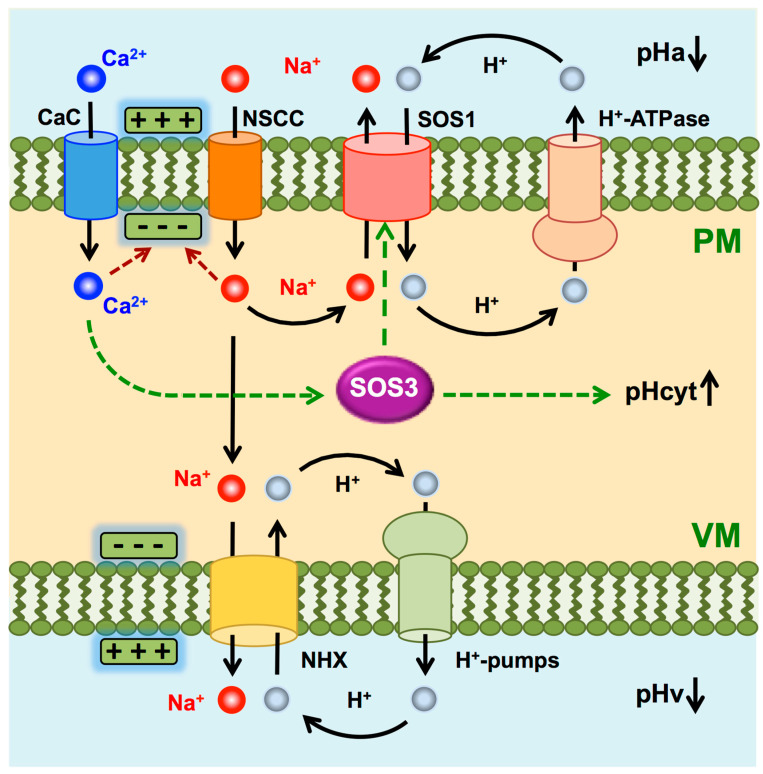
Model of ion transport processes relevant to salinity-induced shifts in pHcyt. For simplicity, only the salt-induced Ca^2+^ entry, Na^+^ fluxes, and H^+^ cycles are shown. K^+^ and Cl^−^ fluxes are omitted. Under salinity stress, Na^+^ and Ca^2+^ entry elicit depolarization of the PM, which activates the H^+^-pump to repolarize the membrane. Intracellular Na^+^ is extruded by SOS1 at the expense of the H^+^ gradient created by the H^+^-pump. Similar fluxes take place at the tonoplast for Na^+^ compartmentation in the vacuole. The enhanced proton-motive force created by pumps at both membranes should be of sufficient magnitude to energize Na^+^ transport with the predicted result of cytoplasmic alkalinization. However, this salt-induced rise in pHcyt was not observed in the *sos3-1* mutant, suggesting that the operation of the SOS pathway is necessary to create the alkalinization response. The Ca^2+^ entry activates SOS3 that in turn promotes Na^+^/H^+^ exchange by SOS1. Salt-induced alkalinization is not a symptom of disturbed ion homeostasis but the consequence of the activation of the SOS pathway. PM: plasma membrane; VM: vacuolar membrane; CaC: Ca^2+^ channel; NSCC: non-selective cation channel; NHX: Na^+^,K^+^/H^+^ exchanger at the tonoplast; H^+^-pumps: V-ATPase and pyrophosphatase; pHa: apoplastic pH; pHcyt: cytosolic pH; pHv: vacuolar pH. Green boxes with +/− symbols represent the electrical membrane potential; dashed green and red arrows mean positive and negative effects, respectively.

Different signaling pathways are activated by salinity-induced Ca^2+^ influx, among which the SOS pathway is paramount for salt tolerance [12,54,59,70]. The activity of SOS1 extrudes Na^+^ in exchange for H^+^, which results in cytosolic acidification. However, salt-induced membrane depolarization also elicits K^+^ loss through depolarization-activated K^+^ outward rectifying channels expressed in roots (e.g., GORK) and enhances P-type H^+^-ATPase activity, both responses aiming to repolarize the membrane [71,72,73]. In single cells, H^+^-pumping and Na^+^/H^+^ exchange would have opposite effects on pHcyt upon salinity treatment (Figure 11). Our results suggest that the net balance of the opposing forces of H^+^-pumping and Na^+^/H^+^ exchange in the meristematic and elongation zone (MEZ) is shifted towards pHcyt alkalinization, signifying that H^+^ efflux by H^+^ pumps predominates over Na^+^ efflux and H^+^ re-entry. Moreover, a proton motive force across the plasma membrane of greater magnitude than the inward-driven Na^+^ gradient is required to energize the electroneutral Na^+^/H^+^ exchange mediated by SOS1. It should be expected that, in the long term, homeostasis is restored and H^+^ efflux and pHcyt return to pre-stress values. We did not observe that restoration within the 40 min time frame of experiments focused on the responsive MEZ sector. In experiments monitoring the whole root after 16 h of salt treatment, differences at the MEZ were difficult to quantitate, but the strong signal regarding the relative alkalinization of pHcyt in the vasculature indicated that the root system had not returned to homeostatic values.

The alkaline pHcyt measured in the vasculature of seedlings exposed to NaCl relative to other root tissues can be explained by the accumulation of Na^+^ in these tissues as the result of the centripetal movement of Na^+^ along the evapotranspiration stream and towards the vasculature, as shown for Arabidopsis, *Thellungiella halophyla*, and rice [54,74,75]. Accumulation of Na^+^ in the root xylem parenchyma would bring about the opposing forces of H^+^-pumping and Na^+^/H^+^ exchange explained above (Figure 11), resulting in cytosolic alkalinization. Again, it should be emphasized that our results do not exclude cytoplasmic alkalinization of epidermal and cortex cells because that tendency could have been obscured by the much greater Na^+^ accumulation in tissues surrounding the vasculature, since Na^+^ loading in the xylem vessels seems to be the rate-limiting step in Na^+^ export out of the root [53,74,75]. In any case, it is worth noting that the relatively alkalinized regions of roots exposed to NaCl, such as the meristematic cells and the vasculature, coincide with the expression pattern of *SOS1* [53], and are therefore expected to correspond to cells and tissues that will be acutely exposed to and protected from Na^+^ toxicity by SOS1.

The observation that no differences were observed between the pHGFP-LTI6b and pHGFP-VTI11 Arabidopsis lines except a small delay in the response of pHGFP-VTI11 relative to pHGFP-LTI6b could mean that either the cytosolic pH variations occurred in all the intracellular space, or that transport activities of each membrane generate the same effect in the cytosol. At the tonoplast, two main proteins are believed to have the capacity of affecting the cytosolic pH: the V-ATPase, whose activity translocating H^+^ into the vacuolar lumen alkalinizes the cytosolic pH, and tonoplast-localized NHXs, whose cation/H^+^ exchanger activity acidifies the cytosolic pH (Figure 11). In Arabidopsis, the predominant vacuolar isoforms AtNHX1 and AtNHX2 should be fully active at tissues under pressure from high Na^+^ concentration [76,77]. In other words, the activity of these vacuolar exchangers would be maximal in conditions demanding greater capacity of cation compartmentation into vacuoles, which in turn could impose a greater demand for proton pumps and contribute towards cytosolic alkalinization. As discussed above, the interplay of H^+^-pumps and SOS1 at the plasma membrane would have the same output, with the pumps alkalinizing the cytosol and SOS1 decreasing pHcyt.

A key question is whether the salt-induced shifts in pHcyt are simply the symptom of disturbed ion homeostasis and the “automatic” operation of the transport systems involved (Figure 11), or if they are instead the result and an integral part of the ”active” response mechanism. In other words, are changes in pHcyt only the consequence of salt stress, or are they a signal produced by the pertinent sensory and response mechanisms? In this regard, the finding that salt-induced pHcyt changes were largely dampened in the *sos3-1* mutant strongly suggests that the pHcyt-shift results from the sensory and response activity of the SOS pathway. Equal NaCl treatments result in a more severe stress in the *sos3-1* mutant root than in the wild-type [14,78]. Consequently, a greater cytoplasmic alkalinization should be expected if that were produced only by Na^+^-induced ionic disturbance. This cytoplasmic alkalinization would arise from the activation of proton pumps by salinity-induced membrane depolarization together with the faulty activation of the Na^+^/H^+^ antiporter SOS1 [17,52,79,80]. Moreover, the PM H^+^ pumps are themselves a target of the SOS pathway in Arabidopsis roots, and the *sos1* mutation affects H^+^ transport even in the absence of salt stress [81]. Hence, we propose that salt-induced cytoplasmic alkalinization is not just a consequence of rebalanced ion transport for Na^+^ efflux, but instead this salinity-induced pHcyt shift is likely to be part of the sensory and response system for which the SOS3 function is required. We also argue that salt-induced changes in pHcyt are informative to plant cells to discriminate between environmental insults, all resulting in Ca^2+^ spikes, which may or may not alter the cellular ionic homeostasis [32,34,82].

The root cross-section pHcyt values in WT seedlings (Figure 5 and Figure 6) show not only the differential alkalization of the vasculature and the epidermis in control and saline conditions, but also that the response to the salt treatment was not homogeneous along the longitudinal root axis. For seedlings grown in control medium, the epidermis and cortex had cells with a more alkaline pHcyt relative to cells in the stele, and this profile did not differ between the four segments analyzed. By contrast, seedlings treated with 100 mM NaCl exhibited an inverted pHcyt pattern, in which the pHcyt increased in the vasculature relative to the external layers of the root, and this response occurred in both reporter lines pHGFP-LTI6b and pHGPF-VTI11 (Figure 5 and Figure 6). The most significant increase in pHcyt took place in the vasculature of zone S2. SOS1, whose activity is controlled by the kinase complex SOS2-SOS3, which plays a fundamental role in Na^+^ fluxes at the vasculature promoting Na^+^ loading in the xylem for long-distance transport in several plant species [53,54,74,83,84,85]. Again, the increase in pHcyt in the vasculature is inconsistent with enhanced Na^+^/H^+^ exchange for Na^+^ extrusion and instead could reflect enhanced H^+^-pumping to energize Na^+^ loading in the xylem or depolarization-induced K^+^ and H^+^ leaks, but the largely reduced response in *sos3-1* seedlings relative to WT plants (Figure 9 and Figure 10) strongly suggests that this phenomenon results from a signaling and response mechanism in which SOS3 plays a substantial role.

Recently, a ”sodium-sensing niche” (SSN) was proposed to operate in a cell group within the early differentiation zone of Arabidopsis roots [16]. The amplitude of the primary Ca^2+^ signal at the SSN and the speed at which the resulting Ca^2+^ wave propagated bidirectionally were both dose-dependent and specifically responsive to Na^+^ ions. The Ca^2+^ sensors SOS3/CBL4 and CBL8, both able to activate the SOS2-SOS1 module, were responsive by distinct Ca^2+^-signal amplitudes. Whereas the SOS3-SOS2-SOS1 module conferred basal salt tolerance, the CBL8-SOS2-SOS1 module became activated only in response to severe and durable salt stress. Accordingly, seedlings lacking SOS3 were sensitive to salt stress levels that produced only moderate Ca^2+^ signals, and the SOS3 protein was less responsive to Ca^2+^-induced structural changes than CBL8 [16]. The signaling activity of the SSN was not analyzed in the *sos3*/*clb4* and *cbl8* mutants to determine to what extent these signaling intermediaries influenced the response. Our data regarding the response of the MEZ, which does not coincide with the SSN in the early differentiation zone, indicate that SOS3 activity is critical for the salinity response of the root. Moreover, in whole-root imaging, salinity-induced alkalinization was also observed in the early differentiation zone possibly corresponding to the SSN (see Figure 5C and Figure 6C). Salinity-induced alkalinization in root sectors S2 and S3 of the wild-type seedlings was largely absent in the *sos3-1* mutant (Figure 9).

The Ca^2+^ spikes and waves triggered by salinity stress have been amply characterized [8,21], but the question remains of whether a Ca^2+^ signature conveys sufficient stimulus-specific information to mount a salinity stress response per se, or whether additional inputs must be perceived, processed, and integrated with the Ca^2+^ signal [20]. The interdependent Ca^2+^-ROS waves travelling acropetally in Arabidopsis seedlings whose root tips are exposed to NaCl mainly operate in the long-distance propagation of the signal [8], but again it remains unclear whether these signals convey the pertinent information to detect the disturbance of cellular ion homeostasis resulting from the ionic component of salinity stress. We hypothesized that because Na^+^ influx depolarizes the plasma membrane and promotes K^+^ efflux [17], and Na^+^ extrusion requires commensurate rates of H^+^ translocation, salt-induced changes in pHcyt could assist the cell in rightly perceiving salinity stress, as opposed to other insults triggering Ca^2+^ signals. Indeed, other stimuli that trigger Ca^2+^ signals coincide with a cytosolic acidification rather than an alkalinization [33,34]. The SSN identified by [16] occurred in the early differentiation zone, which is upstream to the MEZ sector we identified here as a focal point highly responsive to NaCl treatment with regard to changes in pHcyt. However, salinity-induced alkalinization was also observed in the vasculature axis in the S2-S3 sectors comprising the SSN (Figure 5 and Figure 6). This correspondence strongly suggests that both signals, Ca^2+^ transients and shifts in pHcyt, could inform the cell of a sodicity stress. It will be important to investigate whether changes in cytosolic Ca^2+^ and pH occur simultaneously or sequentially by using a dual-reporting sensor [34].

## 4. Materials and Methods

### 4.1. pHGFP Reporters

For this study, we selected the genetically encoded ratiometric pH-sensor pHGFP [37]. Constructs for the stable expression in *Arabidopsis thaliana* of pHGFP-VTI11 and pHGFP-LTI6b translational fusions, both driven by the *UBQ10* gene promoter, have been described elsewhere [38,39]. Localization of the chimeric proteins pHGFP-VTI11 in the tonoplast and of pHGP-LTI6b in the plasma membrane have also been demonstrated prior to this study [38,39] and were confirmed via confocal fluorescence microscopy of roots of transgenic seedlings.

### 4.2. Plant Transformation and Reporter Lines Selection

Wild-type *Arabidopsis* Col-*gl1* and the congenic *sos3-1* mutant [86] were used in this work. Transgenic Arabidopsis plants were produced by *Agrobacterium tumefaciens*-mediated transformation and floral dipping [87]. *A. tumefaciens* GV3010 (Rif^R^, pMP90, pTiC58DT-DNA) was used for transformation of *Arabidopsis thaliana*. When needed, rifampicin at 50 mg/L, gentamicin at 20 mg/L, or kanamycin at 50 mg/L were added to cultures. In all plant transformations made for this study, several primary transgenic lines were tested for coherent pHGFP fluorescence, and representative homozygous lines of the T_3_ generation were used for further experimentation.

For routine plant propagation, seeds were sterilized, stratified (darkness and 4 °C) for 2–4 days, and sawn on pots with Compo^®^ Sana Universal substrate in a plant growth chamber under a long day regime (16 h day/8 h night, 23/19 ^o^C, 60–70% relative humidity), and 250 μmol m^−2^ s^−1^ photosynthetically active radiation (PAR). For pHGFP imaging, seedlings were grown in LAK medium, which is nominally free of Na^+^ and K^+^ [76], supplemented with 1 mM K^+^ and 10 mM MES pH 5.6.

### 4.3. Fluorescence Imaging of pHGFP Reporter Lines

Two different experimental set-ups were used to monitor variations of pHcyt in whole roots or sectors therein. The root imaging procedure to create pHcyt heat-maps followed the method reported by [51] with modifications (Supplemental Figure S1). In brief, *A. thaliana* Col-0 plants expressing the pHGFP fluorescent reporters were grown vertically in LAK medium with 1 mM KCl and 10 mM MES pH 5.6 in a growth chamber. Three-day-old seedlings were transferred to microscope glass-bottom dishes (MatTek, USA) with the same medium and 0.7% low-melting-point agarose. Seedlings were incubated vertically overnight in a growth chamber. Before imaging, seedlings were placed horizontally and 90 μL of liquid LAK medium was added. Seedlings were gently pressed back into their agarose bed and incubated for 40–60 min for recovery. Imaging was performed with a Leica SP5 using a ×10 objective (HC PL Fluotar ×10/0.3 DRY). The pHGFP reporters were excited sequentially with 405 and 488 nm light wavelengths, and emission was detected in a 500–550 range using a HyD2 detector. Frames were acquired every 6 s for 40 min. After 4 min of imaging (stack 40), 10 µL of stock solutions for imposing the different treatments were added on the side of the root, and imaging acquisition continued.

The fluorescence images were processed using Fiji software [88]. The processing steps were: background subtraction, Gaussian blur (1), 32-bit conversion, thresholding, ratio calculation, and royal look-up table. Quantitative imaging data were obtained from processed 32-bit images. Entire images were quantified for presentations of global responses. Heat-maps were generated from 64 adjacent regions of 16 × 178 pixels (24.2 × 268.2 μm). For heat-maps, normalized datasets (Δ*R*:*R*) were calculated as (*R*−*R*0)/*R*0, where *R*0 represent mean 4 min baseline values. Normalized heat maps were obtained from registered movies and each region was normalized to its respective baseline. Graphs and heat-maps were generated using OriginPro software. The final data obtained correspond to the ratio I_405_/I_488_ (intensity of the fluorescence emission after sequential excitation with the 405 nm and 488 nm wavelength, respectively), corrected according to Waadt et al. (2017) and presented graphically as a heat-map representing the pH variation along the root with time. A region between the maturation and elongation zones of the roots produced the strongest pHcyt variations. This behavior was observed in almost all the roots analyzed, independently of the treatment used. For that reason, the numerical data analyzed were taken from this selected zone comprising the meristematic and elongation zones (MEZ segment; 750 µM upwards from the root tip) to avoid the possible masking of cytosolic pH variations by less responsive zones of the root.

For the pHcyt imaging in whole seedlings, plants expressing the pHGFP fluorescent reporters were grown on LAK medium (1 mM KCl, 10 mM MES-KOH, pH 5.6). Four-day-old seedlings grown vertically in the growth chamber were transferred to new plates, supplemented with the same media or different concentrations of NaCl, and grown for an additional 24 h. Microscopy analyses were performed using a Leica TCS LSI microscope equipped with a PLAN APO ×5.0 macro-objective (Leica Microsystems, Wetzlar, Germany). The pHGFP reporters were sequentially imaged with excitation wavelengths of 405 nm and 488 nm and an emission wavelength of 500–550 nm. Seedlings were directly imaged from the plate without any manipulation. To image entire seedlings in X, Y, and Z, Z-stacks at multiple positions were acquired. Image processing and analysis was performed using Fiji. Fluorescence intensity values of Z-stacks were summed up and individual tiles were stitched together using the Grid/Collection stitching tool [89]. After background subtractions, ratiometric image calculations proceeded as described by [90].

To quantify and compare differences in whole-root responses between contrasting genotypes (Col-0 and *sos3-1*) with statistical robustness, the whole seedling imaging protocol was modified to overcome the technical limitations of a small number of samples, long processing time, and limited repetitiveness between individual seedlings. This approach, termed the *One Shot per Root* method (Supplemental Figure S1), allowed the simultaneous imaging of several seedlings, thereby improving the statistical analyses while minimizing sample-to-sample experimental noise. Wild-type and *sos3-1* seedlings expressing the cytosolic pHGFP-LTI6b reporter were grown vertically in plates with LAK medium in a growth chamber. Four-day-old seedlings were transferred to small dishes filled with fresh liquid LAK medium (8–9 seedlings per genotype and treatment) and incubated for recovery at room temperature for 30 min. Next, seedlings were transferred to new dishes with liquid LAK medium supplemented with different concentrations of NaCl and incubated for additional 20 min. After the incubation period and before imaging, seedlings were placed horizontally in a microscope dish and the roots were covered with the same media as the one they had been incubated with. The pHGFP reporters were imaged in a Leica TCS SP5II after sequential excitation with 405 and 488 nm light wavelengths, and emission was detected in the 500–550 nm range using the HyD2 detector. One picture per root was acquired with a 63× objective, comprising the most responsive zone in the meristematic and elongation zones (MEZ) of the root. Images of each root were processed with Fiji software and the fluorescence data were plotted using OriginLab software. Statistical analyses were performed using the OriginPro program.

## 5. Conclusions

We show that salinity stress elicits a rapid cytosolic alkalinization of cells in the meristematic and early elongation zone of the root. Within hours, salinity stress also results in the alkalinization of cells in the root stele around the early differentiation zone, which overlaps with the sodium-sensitive niche recently described to initiate a salt-induced Ca^2+^ wave. Both pHcyt responses were largely attenuated in the *sos3-1* mutant root, strongly suggesting that salt-induced changes in pHcyt are not simply the consequence of disturbed ion homeostasis but instead result from the acclimation response involving the operation of the SOS pathway. We also suggest that these changes in pHcyt may constitute a signal informing cells of salt-affected roots to recognize specific hallmarks of sodicity stress.

## Data Availability

Data contained in this article is part of the PhD dissertation of author Belén Rombolá-Caldentey, which can be found at https://hdl.handle.net/11441/83771.

## Supplementary Materials

The following supporting information can be downloaded at: https://www.mdpi.com/article/10.3390/ijms24043549/s1.
