# Supplementary material for: Salinity-Induced Cytosolic Alkaline Shifts in Arabidopsis Roots Require the SOS Pathway
Source: Int J Mol Sci. 2023 Feb 10;24(4):3549. doi: 10.3390/ijms24043549 (PMC9960406; doi:10.3390/ijms24043549)
Supplement: Supplementary file 1 [file ijms-24-03549-s001.zip › ijms-2164402-supplementary.pdf]

SUPPLEMENTAL MATERIALS

Salinity-induced cytosolic alkaline shifts in Arabidopsis roots require the SOS pathway

Belén Rombolá-Caldentey[1], Zaida Andrés[2,a], Rainer Waadt[2,b], Francisco J. Quintero[1], Karin Schumacher[2], José M. Pardo[1,*]

1. Institute of Plant Biochemistry and Photosynthesis, Consejo Superior de Investigaciones Científicas and Universidad de Sevilla, 41092 Seville, Spain
2. Centre for Organismal Studies, University of Heidelberg, 69120 Heidelberg, Germany

a. Current address: Syngenta Crop Protection AG Research Center, 4332 Stein, Switzerland
b. Current address: Institute of Plant Biology and Biotechnology, University of Münster, 48149 Münster, Germany

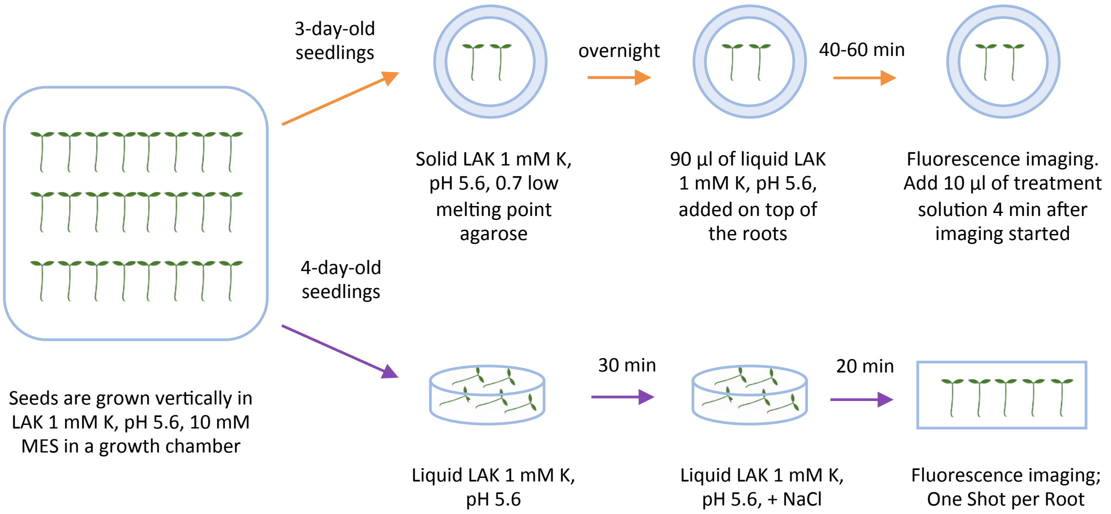

Supplemental Figure S1. Procedure to measure pHcyt changes in Arabidopsis roots. Graphical representation of steps, times and buffers used to measure pHcyt in seedling roots. Upper part, method used to create heat-maps of MEZ. Lower part, the One-Shot per Root imaging method.

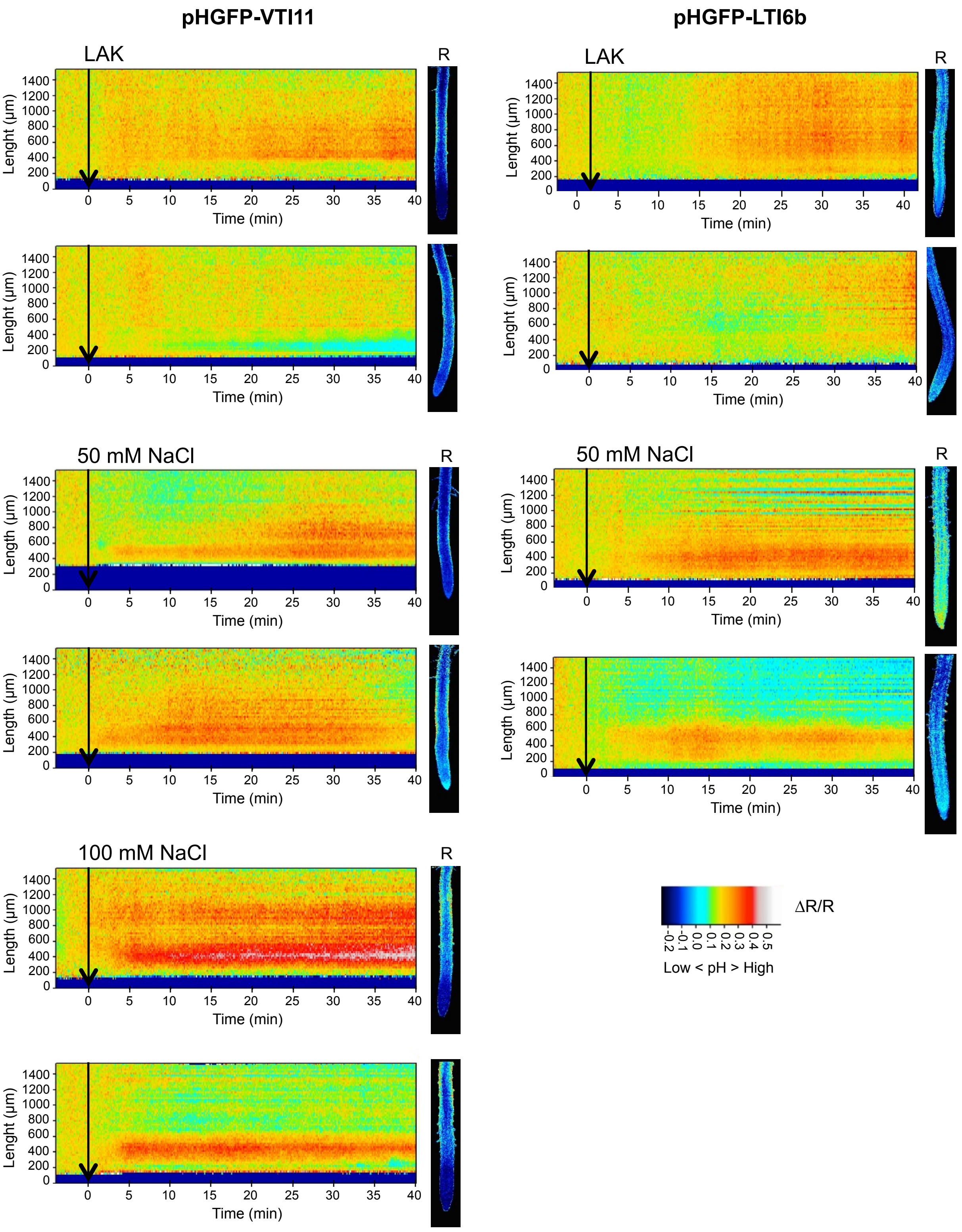

Supplemental Figure S2. Representative heat-maps of seedlings expressing the pH sensors pHGP-VTI11 (left row) and pHGFP-LTI6b (right row) treated with LAK medium supplemented or not with the indicated NaCl concentrations. Heat-maps show normalized data derived from 64 adjacent regions (268.2 x 24.2 µm). Arrows indicate the beginning of the treatment. Small panels next to heat-maps show fluorescence emission ratio (R) images at 35 min time point.

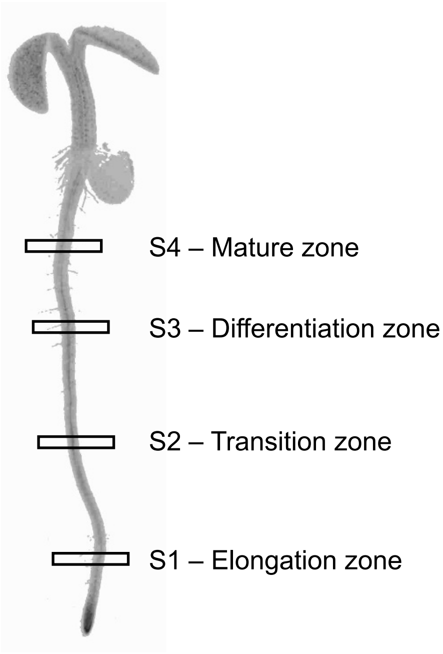

Supplemental Figure S3. Schematic diagram of the root the sectors used to analyze the pHcyt variations along the complete root of *Arabidopsis thaliana* lines expresing the pHGFP reporters. Sectors were named S1-S4 according to the the developmental stage and position from the root tip.

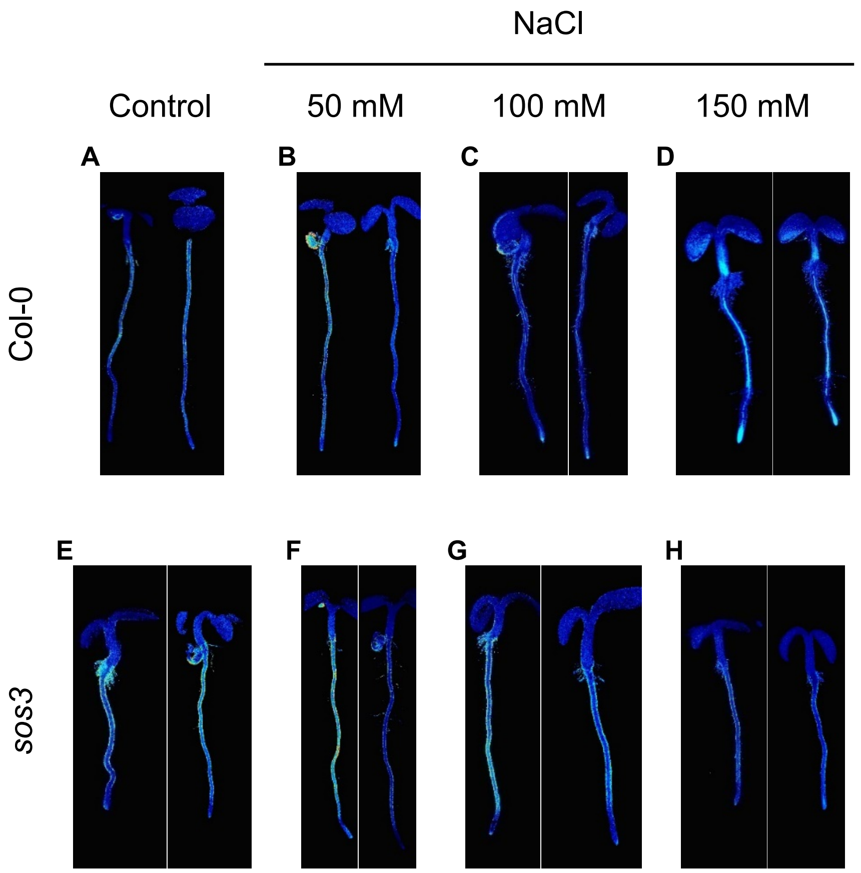

Supplemental Figure S4. Whole seedling pH-maps under salt stress of wild-type Col-0 and *sos3-1* lines expressing pHGFP-LTI6b. Emission ratios (R) of pHGFP-LTI6b in 4-day-old seedlings 24 h after being transferred to LAK medium supplemented with NaCl as indicated. Shown are representative whole seedlings of Col-0 **(A-D)** and *sos3-1* **(E-H).**
